# Perceptions of Conflict of Interest Disclosures among Peer Reviewers

**DOI:** 10.1371/journal.pone.0026900

**Published:** 2011-11-02

**Authors:** Suzanne Lippert, Michael L. Callaham, Bernard Lo

**Affiliations:** 1 Department of Emergency Medicine, Alameda County Medical Center, Oakland, California, United States of America; 2 Department of Emergency Medicine, University of California San Francisco, San Francisco, California, United States of America; 3 Annals of Emergency Medicine, American College of Emergency Physicians, Irving, Texas, United States of America; 4 Department of Medicine, University of California San Francisco, San Francisco, California, United States of America; Johns Hopkins Bloomberg School of Public Health, United States of America

## Abstract

**Background:**

Disclosure of financial conflicts of interest (COI) is intended to help reviewers assess the impact of potential bias on the validity of research results; however, there have been no empiric assessments of how reviewers understand and use disclosures in article evaluation. We investigate reviewers' perceptions of potential bias introduced by particular author disclosures, and whether reviewer characteristics are associated with a greater likelihood of perceiving bias.

**Methodology/Principal Findings:**

Of the 911 active reviewers from the *Annals of Emergency Medicine*, 410 were randomly selected and invited to complete our web-based, 3-part survey. We completed descriptive analysis of all survey responses and compared those responses across reviewer characteristics using 2×2 analyses and the Fisher exact test. We had a response rate of 54%. The majority of reviewers surveyed reported a high level of skepticism regarding financial relationships between authors and industry without a clear or consistent translation of that skepticism into the self-reported actions that characterize manuscript assessment. Only 13% of respondents believed physician consultants authoring articles based on company data are likely to have unlimited data access. 54% believed that bias most likely exists with any honorarium, regardless of monetary amount. Between 46% and 64%, depending on the type of financial relationship disclosed, reported that their recommendation for publication remains unchanged. Respondents reporting personal financial ties to industry were less likely to perceive bias in industry relationships and less likely to believe that bias exists with any monetary amount of honoraria.

**Conclusions:**

We recommend that the monetary amount of all financial relationships be reported with manuscript submissions, lead authors certify that they have unrestricted access to data, and reviewers disclose any financial ties to industry whether or not they are related to the manuscript under review. Further research is required to better understand reviewers' perceptions of financial relationships between authors and industry in order to develop clear and consistent guidelines for incorporating the perception of potential bias into manuscript assessments.

## Introduction

Disclosure of physicians' financial relationships with pharmaceutical companies is a cornerstone of conflict of interest (COI) policies [1]. Media revelations of financial COI in clinical researchers, as well as U.S. Congressional investigations, have highlighted the need to deal with such conflicts. Disclosing authors' COI is intended to help reviewers, editors, and readers assess the likelihood of bias and the potential impact on the validity of the research results and conclusions. For this intention to be manifest, the scientific community expects that reviewers and readers interpret disclosures accurately and consistently, and that they translate their interpretations into an ability to recognize and compensate for potential bias. These assumptions of peer reviewers in particular, however, have not been assessed empirically. A better understanding of how peer reviewers incorporate disclosures of financial conflicts into the assessment of manuscripts that they review could improve the peer review process and thus enhance the validity of research publications.

We conducted a survey as an initial step in investigating how journal peer reviewers interpret various financial relationships between industry and authors and how their interpretations influence their approach to manuscripts. Our research questions were: How do peer reviewers interpret disclosures that an author serves on a speakers bureau or is a consultant for industry? Do reviewers view those roles as sources of potential bias for authors, and to what degree? How do reviewers incorporate their perceptions of those roles into reviews of submitted manuscripts? Are reviewers' personal ties to industry or the number or quality of their reviews associated with a greater likelihood of perceiving bias or altering the appraisal of manuscripts?

## Methods

Our study was conducted at *Annals of Emergency Medicine*, which ranks in the top 11% among 6,620 science and medical journals by number of citations and is the leading emergency medicine journal by impact factor [2]. The vast majority of the papers submitted for review by the academic clinicians and clinical researchers that make up the reviewer pool are original clinical research. *Annals* conducts double-blinded reviews and requires COI disclosures for authors, reviewers, and editors as recommended by the International Committee of Medical Journal Editors (ICMJE) (http://www.icmje.org/ethical_4conflicts.html; http://www.annemergmed.com/content/instauth#conflict). Each review receives a quality rating from the editor supervising the manuscript using a single, global, 5 point scale [3]. This quality rating is virtually identical to the rating scale validated at the British Medical Journal [4], and its reliability has been previously reported [5]. The rating scale has also been found to moderately correlate (R = 0.53) with a reviewer's ability to detect deliberate errors in a test manuscript [5].

We developed a 29-question, 3-part, web-based survey, administered through surveymonkey.com. The initial survey was reviewed and tested with editorial colleagues, who also have extensive experience as reviewers, after which final revisions to the content were made for clarity and convenience. The responses to the survey questions were confidentially linked to the *Annals* database, which tracks reviewer experience and quality rating for all reviews over the past 14 years. The initial questions were designed to assess baseline reviewer knowledge and perceptions of the activities and benefits of two specific potential conflicts commonly disclosed: serving on a speakers bureau and acting as a consultant. In the second portion of the survey, we elicited reactions to hypothetical manuscripts, which included either one of these two COI disclosures or disclosures such as stock ownership, direct financial payments and research sponsorship. Finally, we gathered information regarding participants' personal ties to industry, experience in peer-review, and teaching roles (Survey instrument Appendix A).

### Participants

We invited a sample of 410 reviewers randomly selected from the database of 911 *Annals of Emergency Medicine* active reviewers, those completing at least one review in the past two years. As our objective was primarily descriptive, we did not undertake power calculations. Selected reviewers were contacted by email with a request to participate in a survey evaluating the role of financial disclosures in the peer-review process. Participants were informed that we would associate survey responses with data from the *Annals* files on reviewer experience, coding the associated data for anonymity. Emails were sent to each potential participant at the address they provided for journal reviews; if no response was received, two further attempts were made to contact the recipient while confirming that a correct email address was being used.

### Analysis

Our planned analysis was primarily descriptive. We present the frequencies of responses on a five-point Likert scale, condensing the scale into positive (very likely and likely), negative (unlikely and very unlikely), and don't know responses where possible for simplicity.

We compared respondents to non-respondents and to all Annals reviewers in terms of reviewer age, gender, years since completion of residency, number of reviews completed and reviewer quality rating, as defined above. We gathered professional characteristics of the reviewers, including years since residency, hours per week of didactic teaching, reviewer quality rating, and total number of reviews completed. We evaluated whether these characteristics were associated with an increased frequency in reviewers' perception that a percent income threshold for bias exists; reviewers' knowledge of the activities expected of speakers bureau members and consultants; reviewers' interpretation of the likelihood of bias associated with guarantees of future collaborative projects, sponsorship of research, stock options or direct financial payments; and reviewers' self reported incorporation of disclosures into article assessment (reading more carefully, change in perception of the article's credibility, change in likelihood to recommend for publication). Finally, we examined whether reviewers with financial ties to industry, defined as those who receive any honoraria, work as a consultant, or own stock or equity, are more likely to endorse a potential for bias in industry-related financial ties. Participants who responded, “don't know,” were not included in the 2×2 analyses.

We used SAS 9.2 (SAS Institute Inc, Cary, NC, USA) to calculate Chi square statistics or, where necessary due to sample size, Fisher exact tests, setting a significance level of 0.05. We did not apply the Bonferoni correction for multiple comparisons. IRB approval from UCSF was obtained and each participant was asked explicitly for consent before accessing the survey questions.

## Results

Of the 410 invited reviewers, eight were excluded because the original email address was not correct and could not be validated. 218 reviewers completed responses to the survey (response rate of 54%). Table 1 shows the characteristics of the sample; there were no differences between respondents and non-respondents [data not shown].

**Table 1:** Participant characteristics.

Descriptor	Total n	
Male	217	151 (69%)
Mean Age, years	176	48 (SD 8.4, range 28–69)
Median years since completion of residency	183	18 (range 0–40)
Median number of reviews completed	194	11 (range 1–80)
Mean reviewer rating (5 point scale, 5 outstanding)	194	3.8 (SD 0.62)

### Respondent perceptions of pharmaceutical company speakers bureaus

**Figure 1:**
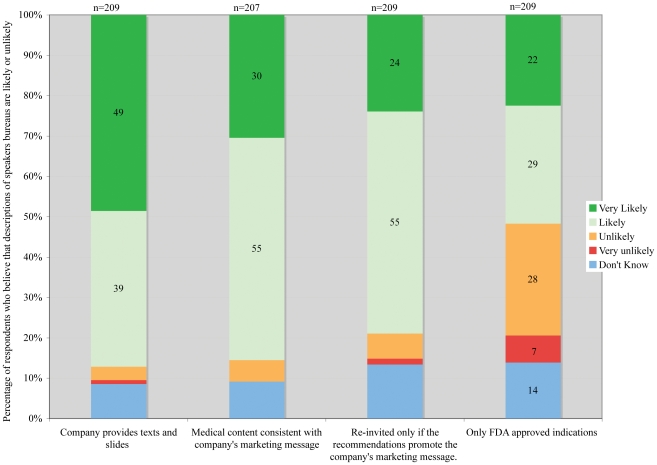
Respondent perceptions of arrangements in pharmaceutical company speakers bureaus.

A large majority of respondents believed that companies exert various types of influence over the content of lectures given by physicians on the company's speakers bureau, including provision of text and slides (88%), consistency of the medical content with marketing messages (85%), and selective re-invitation of speakers (79%) (Figure 1). Respondents without financial ties to industry (as defined in our methods) were significantly more likely than respondents with ties to agree that the content of speakers bureau talks is consistent with the company's marketing message; (99% vs. 88%, Fisher's exact test p = 0.01).

The FDA requires that talks sponsored by a drug company mention only FDA-approved uses. However, 35% of respondents thought it unlikely that a company must ensure that speakers bureau presentations mention only FDA-approved indications, while an additional 14% did not know. More reviewers without financial ties than those with ties thought it unlikely that the company must make that assurance (51% vs. 34%, p = 0.04, Fisher's exact).

### Respondent perceptions of consultant activities for pharmaceutical companies

**Figure 2:**
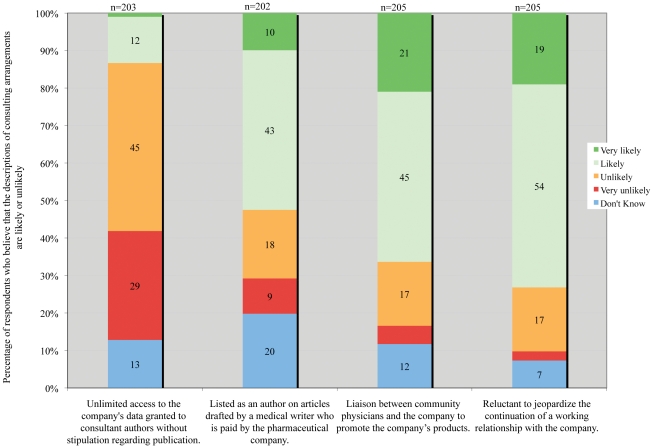
Respondent perceptions of consultant activities for pharmaceutical companies.

A majority of respondents believed that companies exert influences over the content of articles authored by physicians working as consultants (Figure 2). These influences include a belief that consultants have limited access to data (74%), collaborate with company ghostwriters (53%), have goals aligned with the company's marketing message (66%), and are reluctant to jeopardize a future working relationship with the company (73%). Reviewers without any financial ties to industry were significantly more likely to believe that consultants act as a liaison between community physicians and the pharmaceutical company to promote the company's products (84% vs. 68% p = 0.03, Fisher's exact). In addition, significantly more reviewers without ties believed that a reluctance to jeopardize the working relationship is likely (87% vs. 74% p = 0.05, Fisher's exact).

There were no significant differences in reviewers' responses to questions regarding speakers bureaus or consulting based on years since residency, hours per week of teaching, reviewer quality rating, or number of total reviews completed.

### Respondent perceptions of potential bias

**Figure 3:**
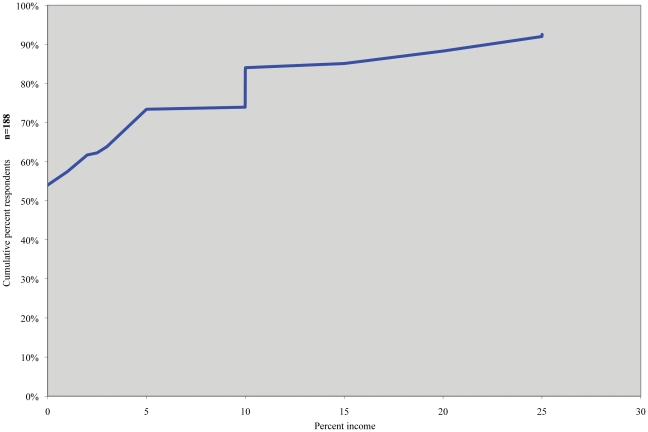
Cumulative percent of respondents who report the threshold for physician income from pharmaceutical companies beyond which an author's judgment is most likely biased. N = 188.

Fifty-four percent of respondents believed that any level of honoraria from a pharmaceutical company, no matter how small, would most likely bias the author's judgment. Respondents without financial ties to industry were more likely than respondents with ties to believe this (67% vs. 33% Fisher's exact p = 0.02). Eighty-four percent of respondents reported that bias was most likely to exist if an author received honoraria totaling up to 10% of professional income (Figure 3). With regard to specific types of consulting relationships, 89% of respondents believed that research support is likely to influence a consultant's judgment when authoring an article, and 67% felt similarly about direct financial payments agreed upon in advance.

### Impact on review of submitted manuscripts

**Figure 4:**
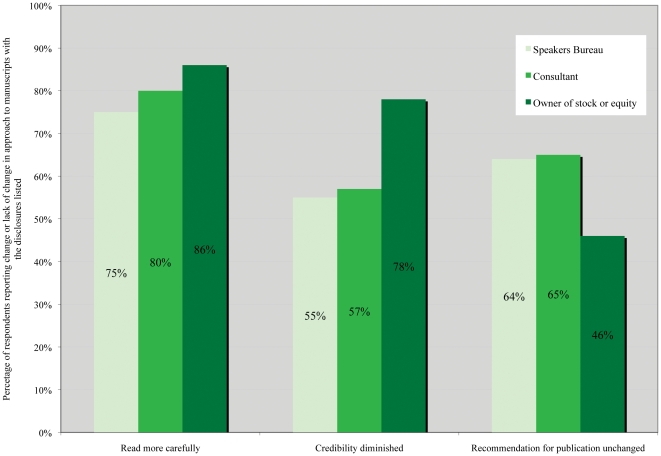
Impact of disclosures on respondents' review of manuscripts.

Ninety-nine percent of respondents reported reading the financial disclosure statement, with 66% reading it before reading the manuscript. The majority of respondents would read a manuscript more carefully and consider the credibility diminished if the lead author disclosed serving on the speakers bureau, acting as a consultant, or owning stock in the company who manufactured the medication being studied. The majority then reported that their recommendation for publication would remain unchanged in relation to those disclosures if no design or statistical flaws were identified (Figure 4).

## Discussion

Because of highly publicized cases of financial conflicts of interest leading to a lack of scientific integrity in published articles [6], journals have been urged to adopt comprehensive COI disclosure requirements for authors [7]. For disclosure to be effective, reviewers must be able to critically assess to what extent a conflict of interest exists, to judge the impact that COI may have on the validity of a submitted manuscript, and to incorporate those judgments into a recommendation regarding publication. In one of the only empirical studies of the influence of COI disclosures on readers' perceptions of an article, Schroter et al [8] completed a randomized control trial in which 900 British Medical Journal (BMJ) readers were provided an article with one of three possible COI disclosures. The “financial statement” declared the authors to be employees or owners of stock,” the “grants statement,” declared the authors to have received research funding and the “none statement” declared no COI. The importance, relevance, validity, and believability ratings of the articles were significantly lower for the article in which “authors are employees and potentially own stock” than in the “none declared” group. Building on these findings but focusing our population of interest on peer reviewers, who are directly involved with decisions regarding the publication of research in contrast to the general readership, we carried out an empirical study with experienced reviewers of medical research from a journal that has an explicit COI policy for authors, reviewers, and editors. We had 4 major findings.

First, the vast majority of respondents perceive of roles on speakers bureaus or as consultants to be aligned with company marketing goals. Even activities condemned by the International Committee of Medical Journal Editors (ICMJE), World Association of Medical Editors (WAME), and Committee on Publication Ethics (COPE), such as ghost writing or authoring articles with only limited access to data, are believed to be likely occurrences [6].

Second, the majority of our respondents did not believe that the bias attributed to these roles has a minimum monetary threshold. Over one-half of respondents believe that bias most likely exists with any honorarium, regardless of monetary amount; while over 90% of respondents believe that bias most likely exists even if authors received up to only 17% of income from a pharmaceutical company.

Third, in contrast to respondents without financial ties, those who disclosed having any personal financial ties to industry were less likely to attribute bias to speakers bureau and consultant roles, and less likely to believe that honoraria of any monetary value, no matter how small, introduces bias (Table 2). There are several possible explanations for this discrepancy between respondents with and without financial ties to industry.

**Table 2:** Comparison analysis of reviewers with or without personal financial ties to industry.

Percentage of reviewers of the total with or without ties responding that the statements below are likely or very likely to be true	N = Total reviewers without financial ties	N = Total reviewers with financial ties	X2	p	Fisher p
Only FDA indications mentioned in physician speakers lectures	80 (49%)	62 (66%)	4.3	0.04	0.04
Company provides the physician speaker with the prescribing patterns of the audience	65 (71%)	49 (51%)	4.6	0.03	0.03
Presentation expected to be consistent with the company's marketing message	82 (99%)	66 (88%)	7.6	0.006	0.01
Consultant provides the company with the names of community physician opinion leaders	82 (98%)	61 (89%)	4.8	0.028	0.04
Consultant acts as a liaison with community physicians with the goal of promoting the company's products	82 (84%)	62 (68%)	5.4	0.03	0.03
Consultant reluctant to jeopardize the continuation of a working relationship with the company.	87 (87%)	66 (74%)	4.3	0.04	0.06
Guarantees of future collaborative projects with the sponsor are likely to bias an author.	89 (93%)	66 (79%)	7.1	0.008	0.01

Respondents who serve on speakers bureaus or act as consultants may have found, in their direct experience, that companies did not expect their lectures to be aligned with marketing goals and that access to data was unlimited. In addition, these reviewers may have more concrete, inside knowledge about the actual activities and benefits of working as a consultant or on a speakers bureau. This interpretation is supported by our finding that reviewers with personal financial ties to industry were more likely to accurately state that companies must ensure speakers bureau presentations mention only FDA approved uses. However, our study can neither confirm nor refute this explanation.

Basic psychological research suggests “that when individuals stand to gain by reaching a particular conclusion, they tend to unconsciously and unintentionally weigh evidence in a biased fashion that favors that conclusion.” [1] Based on this psychological vulnerability to unintentional bias, the discrepancy we found between those with and without personal financial ties to industry may be present because respondents with financial relationships to pharmaceutical companies project their belief that they are immune to undue influence or discount the potential influence in order to prevent cognitive dissonance. This interpretation raises the concern that reviewers with personal ties to industry, regardless of the relevance to a particular article under review, may themselves be vulnerable to minimizing the possible effects of financial relationships with industry to prevent cognitive dissonance when assessing an author's potential of bias.

Finally, most respondents reported that they read articles more carefully and consider the credibility diminished if the author discloses a financial relationship with the manufacturer of the study drug. About two-thirds of respondents would not change their recommendation regarding publication if they found no design or statistical concerns regardless of the author's financial disclosures. Thus reviewers express skepticism of industry relationships but report no overt discrimination against industry-funded manuscripts; rather, their answers suggest that they base their decision for publication on the perceived scientific merit of the manuscript.

Although this is reassuring, it should be interpreted in light of possible social desirability bias as the results rely on self-reported attitudes and behaviors. Additionally, it should be interpreted with an understanding of reviewers' known limitations, including reviewers' variable capacity to find statistical and design flaws within manuscripts under review. In a study using a fictitious manuscript with purposeful errors, 68% of reviewers did not realize that the conclusions of the work were not supported by the results [9]. Similarly, a subsequent investigation reported that reviewers found only an average of three out of nine, major, deliberately induced errors in papers manipulated for the study [10]. Although our reviewers reported reading papers more carefully and critically, it remains unknown whether after reading author disclosures of financial relationships to the study sponsor or drug manufacturer, they are in fact better able to detect flaws in a manuscript and to recommend revisions to mitigate those flaws.

### Limitations

Our response rate was 54% despite multiple attempts to contact potential participants via email. Nevertheless, no significant differences were found between responders and non-responders or between responders and the larger reviewer pool in reviewer experience, reviewer volume, review quality rating, age, or gender. Even in the unlikely situation that all the non-respondents had answered differently than responders, there would still have been a large proportion of reviewers sharing the views reported here.

As with any survey relying on self-reported attitudes and behaviors, stated preferences are highly subject to social desirability bias. Because of the time, effort, and logistics required, recruiting and randomizing reviewers to review fictitious manuscripts that reveal or omit conflict disclosures was not feasible, particularly since no antecedent intermediate studies have been published to date. Such a study of fictitious manuscripts should be considered the next step in research of this topic.

Our survey also relies on questions about the industry-author relationships: speakers bureau member and consultant, which were not further defined in detail. Nevertheless, actual characteristics of speakers bureaus or consulting arrangements are not known, uniform, or found in disclosures required by journals. Ultimately, we expect that the interplay of perceptual influences and the imprecision in disclosures within our survey mirrors those faced by reviewers who are presented with actual conflict of interest disclosures on manuscripts under review. The reported reviewer perceptions, therefore, may be important influences on how reviewers assess submitted manuscripts.

Our findings may not generalize to other journals or specialties. Nevertheless, this particular reviewer population is similar to reviewers from other journals and specialties [5], [11], [12], including the ability of these reviewers to detect deliberate introduced flaws in a manuscript [9], [10], [13] [14], [15], [16], [17].

We did not adjust our levels of statistical significance for multiple comparisons with the Bonferoni correction, which may be too conservative; readers should interpret our findings in light of multiple comparisons we made.

### Conclusions

Based on our findings, we offer several practices that might help reviewers and readers better understand the degree and character of potential bias introduced by financial disclosures:

- **Because there is neither evidence nor consensus to support a specified minimum monetary threshold below which bias does not exist, the monetary amount of all financial relationships should be reported with manuscript submissions.** We recommend that all financial relationships should be disclosed because over one-half of our reviewers believed that any honorarium would most likely bias the author's judgment. Because the percentage of reviewers who held this belief increased as the specific amount of honorarium increased, we recommend that the exact monetary amount also be disclosed.
- **Lead authors should be required to certify that they have had unrestricted access to all data and statistical analysis, and the right to publish in accordance with ICMJE recommendations.** Several recent incidents support the need for adherence to this recommendation. These incidents exposed that important adverse events were not reported in publications, and that several authors received either incomplete data or only the final results tables with no access to the analysis undertaken or original data set [18], [19], [20].
- **Because our results raise the concern that reviewers with personal ties to industry may be vulnerable to minimizing the possible bias associated with financial relationships with industry, all reviewers should disclose any financial ties to industry whether related to the article under review or not.** In support of this recommendation, other studies suggest that individual physicians may be poor judges of whether a financial relationship with industry is relevant to the study at hand [18].

Finally, the majority of reviewers surveyed report a high level of skepticism regarding financial relationships between authors and industry without a clear or consistent translation of that skepticism into actions of manuscript assessment and recommendation. Organizations like ICMJE, COPE, and WAME have all developed increasingly specific and detailed guidelines on COI disclosures over the past 10 years, and exhorted all journals to do the same. Although these increasingly strict and comprehensive disclosure guidelines have been recommended, our results drive us to ask whether disclosure alone truly aids reviewers in identifying potential bias, accounting for the magnitude of its effect and translating that understanding into action. In addition to the greater detail about monetary amounts and actual activities associated with particular disclosures, we propose that research is needed to better identify the components of study design vulnerable to COI bias and the specific components of analysis and result reporting most likely to harbor that bias. Once these components are identified, journals could develop unique guidelines to aid reviewers in identifying bias more consistently, and in more accurately accounting for the effect of that bias in their manuscript assessment.
